# Checkpoint Inhibitors and Therapeutic Vaccines for the Treatment of Chronic HBV Infection

**DOI:** 10.3389/fimmu.2020.00401

**Published:** 2020-03-04

**Authors:** Ruben C. Hoogeveen, André Boonstra

**Affiliations:** Division of Gastroenterology and Hepatology, Erasmus MC, University Medical Center, Rotterdam, Netherlands

**Keywords:** hepatitis B virus, immunotherapy, HBV-specific T cells, checkpoint inhibitors, therapeutic vaccines

## Abstract

Treatment of chronic hepatitis B virus (HBV) infection is highly effective in suppressing viral replication, but complete cure is rarely achieved. In recent years, substantial progress has been made in the development of immunotherapy to treat cancer. Applying these therapies to improve the management of chronic HBV infection is now being attempted, and has become an area of active research. Immunotherapy with vaccines and checkpoint inhibitors can boost T cell functions *in vitro*, and therefore may be used to reinvigorate the impaired HBV-specific T cell response. However, whether these approaches will suffice and restore antiviral T cell immunity to induce long-term HBV control remains an open question. Recent efforts have begun to describe the phenotype and function of HBV-specific T cells on the single epitope level. An improved understanding of differing T cell specificities and their contribution to HBV control will be instrumental for advancement of the field. In this review, we outline correlates of successful versus inadequate T cell responses to HBV, and discuss the rationale behind therapeutic vaccines and checkpoint inhibitors for the treatment of chronic HBV infection.

## Introduction

Hepatitis B virus (HBV) infection is an immense burden to global health. Despite the availability of an effective prophylactic vaccine over 250 million individuals are chronically infected (1). Chronic infection contributes to the development of fibrosis, cirrhosis, and hepatocellular carcinoma, leading to an estimated 887,000 deaths annually (1, 2). Most patients require lifelong nucleos(t)ide analogue (NA) treatment since therapy is not curative, and merely suppresses viral replication. However, NA treatment is highly effective, reduces liver inflammation and has a good safety profile. Still, treatment does not eliminate the risk of hepatocellular carcinoma; and withdrawal from treatment can cause viral relapse and severe inflammation of the liver. Long-term therapy also rarely leads to a functional cure of a chronic infection, as defined by the clearance of the serum hepatitis B surface antigen (HBsAg) and an undetectable viral load in serum. Thus, there is an urgent need to develop new and more effective therapeutics.

**Figure 1:**
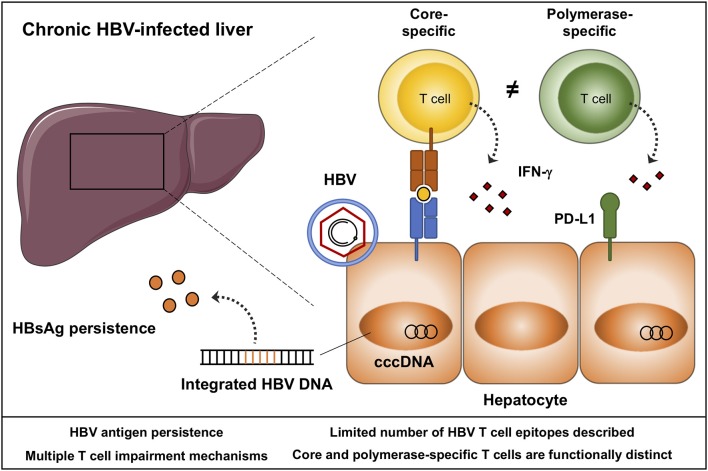
Schematic representation of a chronic HBV-infected liver. HBV persists as covalently closed circular (ccc)DNA in the nucleus of hepatocytes, and integrates into the host genome. Integrated and episomal HBV DNA contribute to ongoing viral antigen secretion, in particular HBsAg. HBV-specific T cells target hepatocytes infected with HBV through the secretion of cytokines and cytolytic molecules. Core and polymerase-specific T cells are functionally distinct. Envelope-specific T cells are typically absent during chronic HBV infection, and with the pre-existing quantities of HBsAg, these cells may be a less suited target to boost by immunotherapy. Aspects that need to be considered in the design and monitoring of HBV immunotherapy are listed.

HBV is a small enveloped DNA virus belonging to the family *Hepadnaviridae*. The virus has a distinct tropism for hepatocytes in which it generates a covalently closed circular (ccc) DNA template to produce new virions, and is able to integrate into the host genome (Figure 1) (3). The persistence of the viral genome represents a major obstacle preventing a sterilizing cure of a chronic HBV infection. It remains unclear if, and how, this transcriptional template can be completely eliminated from all hepatocytes. In addition, transcription of HBV DNA, either from cccDNA or from integrated HBV DNA leads to the secretion of high quantities of viral antigen, predominantly HBsAg (Figure 1) (4), which is thought to hinder effective antiviral immune responses. Nevertheless, permanent containment of HBV replication can be achieved, as HBsAg clearance infrequently occurs following a chronic infection, and is readily observed following acute infection in adults. Central to viral resolution are HBV-specific T cells, that become impaired during chronic infection (5). Restoration of T cell immunity through immunomodulation has become a field of active investigation, with several immunomodulatory strategies being explored in preclinical and clinical studies. The ultimate goal of HBV immunotherapy is to induce a sustained loss of HBsAg transcription while cccDNA persists within hepatocytes. This state mirrors that what is observed in adults following acute HBV infection (6). Loss of HBsAg will not only allow chronic patients to discontinue therapy, it is also associated with lower hepatocellular carcinoma risk, especially if HBs seroconversion occurs before the age of 50 (7, 8).

HBV immunotherapy aims to suppress HBV replication long-term by reinvigorating the host immune response. Immunotherapy can target innate and adaptive immunity. Innate strategies include those that activate pattern recognition receptors, such as toll-like receptors and retinoic acid-inducible gene I (RIG-I), rely on cytokines directed at HBV-infected hepatocytes or activate NK cells. Adaptive strategies consist of therapies that restore T cells and antibodies, including T cell receptor (TCR) redirected T cells, chimeric antigen receptor (CAR) T cells and bi-specific soluble TCRs; checkpoint inhibitors and therapeutic vaccines. In this review we specifically focus on checkpoint inhibitors and therapeutic vaccines since they are capable of enhancing both T cell and B cell immunity. However, also other strategies are being explored, such as the reduction of HBsAg secretion from infected hepatocytes by blocking mRNA transcription by using small interfering RNA (siRNA) that knock down the expression of specific viral transcripts (9, 10).

Experimental data in the chronic lymphocytic choriomeningitis virus (LCMV) mouse model has demonstrated that checkpoint inhibitors and therapeutic vaccines can, to some extent, boost antiviral immunity (11, 12). Whether similar approaches will be sufficient to achieve a functional cure of a chronic HBV infection remains to be seen. In the coming years these strategies will be evaluated in order to determine their safety, efficacy, and optimal dosing. In this context, identifying the patients in which the immune system is amendable to these therapies will be essential. An improved understanding of the immunological mechanisms associated with control of HBV replication will be instrumental for design, and monitoring of these interventions.

Herein, we outline our current understanding of HBV control as can be observed in individuals following an acute or chronic resolving infection with a specific focus on T cells. Furthermore, we provide an overview of the mechanisms and characteristics of HBV-specific T cell exhaustion, and we examine the degree of T cell restoration that can be observed with current therapies. These paragraphs will serve as a framework to discuss the rationale behind immune checkpoint modulation and therapeutic vaccines for the treatment of chronic HBV infection.

## Protective Immunity Following an Acute or Chronic Resolving HBV Infection

HBV infection is self-limiting in over 95% of infected adults. Only a small subset of patients progresses to a chronic stage, and this occurs more often in those patients who are immune compromised (13). The majority of chronic HBV patients are, however, infected early in life, as a newborn or a child. Factors that determine the outcome of infection remain largely undefined but certain human leukocyte antigens (HLA), such as HLA-DPA1 and DPA2 have been associated with an increased risk of a chronic HBV infection (14–16).

There is strong evidence that HBV-specific T cells are required for viral resolution. This is supported by chimpanzee studies in which CD4 or CD8 T cell depletion resulted in failure to control acute HBV infection (17, 18). Furthermore, acute resolving HBV infection is characterized by robust, highly functional T cells directed against all viral proteins. Viral resolution already becomes apparent during the incubation phase as HBV DNA levels start declining even before the onset of symptoms and liver damage (19–21). Clinically, viral resolution is defined as HBs seroconversion and undetectable HBV DNA in serum, which generally occurs within the first 24 weeks post infection. After HBs seroconversion, the frequency of the HBV-specific T cells declines and continues to do so until at least 40 weeks after the onset of symptoms (22). The residual memory T cell response will persist for decades and provide effective containment of HBV replication while the viral genome persists as cccDNA within hepatocytes. The exact mechanisms of T cells driving control of HBV replication are poorly defined, but they are thought to primarily use noncytopathic effector functions (Figure 1) (23, 24).

We and others have recently observed distinct T cell functions depending on the targeted HBV epitope (22, 25, 26). For example, in early acute infection we observed that HLA^*^A2:01 core_18_-specific T cells were characterized by higher expression of molecules, such as granzyme B and perforin, and showed a stronger cytokine response (IFN-γ) following peptide stimulation compared to polymerase_455_-specific T cells from the same individual (22). These findings were accompanied by a distinct T cell phenotype *ex vivo*. Core_18_-specific T cells showed a stronger expression of T cell activation markers (CD38 and PD-1), had increased levels of the T-box transcription factor T-bet when compared to polymerase_455_-specific T cells (27–29). Most HBV-specific T cells were classified as effector memory cells, but polymerase_455_-specific T cells had higher frequencies of effector memory T cells re-expressing CD45RA (TEMRA). These findings suggest a distinct regulation and contribution of epitope-specific T cells to viral control.

Most of our understanding of HBV control is defined in adult acute resolving infection. How these findings translate to chronic patients who clear HBsAg is yet to be determined. These subjects are difficult to study as the estimates of HBsAg clearance rates for NA therapy are around two percent with no differences between HBeAg positive or negative patients [reviewed in (30)]. Additional studies are still needed to determine if HBsAg clearance rates vary among different patient populations and HBV genotypes (31). Recently, a protective effect of core and polymerase-specific T cells against hepatic flares when patients are taken off therapy, was reported (32). This could imply that these specificities are an attractive target for immunotherapy, in particular because these specificities are often detectable during chronic infection (22, 25, 33).

## T Cell Exhaustion During Chronic HBV Infection

In chronic infection, HBV-specific T cells gradually become dysfunctional, and lose their ability to proliferate, produce cytokines, and exert cytotoxicity toward infected cells. This phenomenon, also known as T cell exhaustion, was first described in mice chronically infected with LCMV (34, 35). T cell exhaustion is characterized by a sustained overexpression of multiple inhibitory molecules. Ultimately, severely exhausted T cells are lost through cell death. Chronic HBV-infected patients display many of the hallmarks of T cell exhaustion. Indeed, the frequency of HBV-specific T cells is diminished and even more so in patients with a high viral load (36). The residual T cell response is directed against a limited number of epitopes, primarily located in the core and polymerase proteins, with few responses directed against the envelope and X proteins (25, 33, 37, 38).

Preserved HBV-specific T cells selectively over express several inhibitory molecules, of these PD-1 has been best-characterized (29, 38, 39). Other inhibitory receptors such as LAG-3, TIM-3, and CTLA-4 have not been studied as extensively. Some of these markers are difficult to study because they are often insignificantly expressed on HBV-specific CD8 T cells in peripheral blood (40), but can more easily be detected on T cells within the liver (41). Intrahepatic virus-specific T cells often display a more profound exhausted phenotype, reflected by a lack of the memory marker CD127 and a stronger co-expression of inhibitory receptors, such as PD-1 and TIM-3 (36, 39, 41, 42). The exhausted phenotype of HBV-specific T cells is equally paralleled by functional defects with a reduced cytotoxic, proliferative, and mitochondrial function (36, 38, 43–46). Interestingly, it was found that the function of exhausted HBV-specific CD8 T cells could partially be restored by correcting mitochondrial dysfunction using mitochondria-targeted anti-oxidants (43).

HBV-specific T cell exhaustion is principally maintained by the continued exposure to HBV antigens. PD-1 ligands, suppressive cytokines such as IL-10 and TGF-ß (47–51), impaired function of dendritic cells (DC), natural killer (NK) cells, and increased frequencies of regulatory T cells and myeloid-derived suppressor cells (MDSC), have all been suggested to negatively impact HBV-specific T cell immunity (52–61). The virus can also escape immune pressure through viral escape mutants. HBV has a relatively low mutation rate and because the open reading frames of the viral genome partially overlap, there are constraints to the number of amino acid substitutions that are viable. Nonetheless, genetic diversity consistent with immune pressure is observed during chronic infection: mainly within the core and envelope protein, but also to a lesser degree in the polymerase protein (62–64). In sum, there are multiple mechanisms contributing to T cell dysfunction during chronic HBV infection. This indicates that modulating a single pathway may not sufficiently restore antiviral T cell immunity to attain a functional cure of a chronic HBV infection.

## T Cell Recovery With Current Treatment Regimens

Chronic HBV infection is a highly heterogenous disease characterized by varying levels of viral replication and liver damage. Clinically this has led to the categorization of patients into four clinical phases, based on varying serum levels of HBV DNA, HBeAg, and alanine aminotransferase (ALT). NA treatment is generally only administered in those phases with elevated serum ALT levels, that indicate liver damage resulting from immune-mediated lysis of hepatocytes (65). These phases are best known as immune tolerant (HBeAg positive infection), immune active (HBeAg positive hepatitis), inactive carrier (HBeAg negative infection), and HBeAg-negative hepatitis (65). NA treatment is highly effective in almost all chronic HBV patients leading to an undetectable serum HBV DNA. With respect to T cells, effective NA treatment in HBeAg positive chronic patients has repeatedly been associated with a partial and transient recovery of HBV-specific T cells, with increased proliferation and function *in vitro* (66–68). Recovery of T cell function has been observed as early as two weeks after start of NA therapy (66, 67), but wanes off after approximately six months of treatment (68). Prolonged NA treatment of HBeAg negative patients also leads to a partial, but more long-lasting, recovery of T cells (69). The partial recovery of T cell function following NA therapy is to some extent remarkable because, although HBV DNA becomes undetectable, these agents generally do not lower serum HBsAg levels (70). The persistence of HBsAg may explain why these HBV-specific T cells remain less functional when compared to patients who clear HBsAg following an acute or chronic infection (69, 71, 72). This suggests that only long-term successful suppression of both HBV replication and antigen production will allow for a more profound recovery of T cell function. On the other hand, studies in the LCMV mouse model and chronic HCV infection indicate that virus-specific T cells remain exhausted, even following the complete eradication of antigen, because of an irreversible epigenetic state (73–76). Therefore, HBV antigen removal should likely be supported by additional immune modulation to achieve a functional cure.

## Immune Checkpoint Blockade to Boost HBV-Specific T Cells

**Figure 2:**
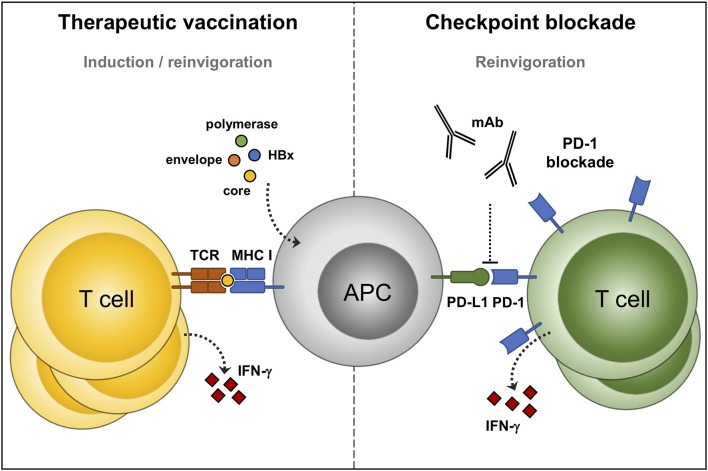
Immunotherapeutic options to reinvigorate defective HBV-specific T cells. Therapeutic vaccines consist of, or express, HBV antigens. Processing of these antigens by professional antigen presenting cells (APC) can prime new, and reactivate pre-existing, HBV-specific T cells (**left panel**). Immune checkpoint inhibitors: monoclonal antibodies that prevent the interaction between programmed cell death protein-1 (PD-1) and its ligand, and boost the function of HBV-specific T cells (**right panel**).

HBV-specific T cells are required for long-term HBV control, but become functionally defective, and greatly reduced in their frequency during chronic infection. Nevertheless, functionally impaired T cells are maintained, making them a potential target for immunotherapeutic intervention. One approach to boost HBV-specific T cells is to prevent the interaction of inhibitory receptors on their cell surface with their ligands. Studies in the chronic LCMV mouse, HBV mouse, and woodchuck model have demonstrated that immune checkpoint blockade can reinvigorate T cell function (11, 77, 78). Similarly, blocking PD-1 (28, 36, 38, 39, 41), CTLA-4 (43), TIM-3 (40, 42), and 2B4 (44) have previously been described to boost HBV-specific T cells *in vitro* (Figure 2). Of these receptors, PD-1 is often the dominant responsive receptor when blocked *in vitro* (39). Checkpoint blockade mainly improves T cell proliferation, and to a lesser degree T cell function. Not all HBV-specific T cells are equally susceptible to checkpoint blockade. Effector memory HBV-specific CD8 T cells from peripheral blood are most responsive to PD-1 blockade, similar to what has been observed for chronic HCV and HIV-infection (39, 79, 80). Intrahepatic virus-specific T cells are often more exhausted than their peripheral counterparts, and therefore benefit from the blockade of additional inhibitory receptors (36, 81). At present, the number of clinical trials evaluating checkpoint blockade in chronic HBV infection are still limited. One of these studies was performed to assess efficacy in a phase 1/2 clinical trial to treat hepatocellular carcinoma, with some patients being infected with HBV, but T cell function was not assessed (82). In another study a group of HBeAg-negative chronic HBV patients received a single low-dose of nivolumab to block the PD-1 pathway (83). This study reported one out of fourteen patients achieving a functional cure, with most patients having a minimal decline of HBsAg. Core and envelope-specific T cells were analyzed by fluorospot, but T cell responses did not change in frequency over time. Both studies included virally suppressed chronic HBV patients so any effect on HBV DNA could not be detected. PD-1 blockade is generally well tolerated at a low dose, but additional dosage studies will be clearly needed to further assess their efficacy and safety since only a few small studies have been conducted. Higher dosages, or combination therapy, could permit a more pronounced recovery of T cells, but simultaneously increases the risk of adverse events, such as autoimmune diseases and hepatic flares (84–86). Further development of checkpoint inhibitors as standard care for chronic HBV infection should clearly take into account their safety profile, since current NA treatment has virtually no side effects and low cost.

## Therapeutic Vaccines

In contrast to checkpoint inhibitors which reinvigorate the function of pre-existing antiviral immunity, therapeutic vaccines are designed to boost immunity by also priming new antiviral responses (Figure 2). Therapeutic vaccines differ from preventive vaccines in their mode of action and in their administration during infection, instead of before infection. Therapeutic vaccines rely on inducing effective CD4 and CD8 T cell responses and not as much on B cells and antibody responses. Moreover, HBV vaccine antigens are preferably presented to the immune system outside the liver allowing processing by professional antigen presenting cells, such as dendritic cells (87). Overall, there is substantial evidence from the LCMV and HBV mouse models supporting the use of therapeutic vaccination for the treatment of chronic viral infections (88, 89). Unfortunately, many of these animal model-derived therapeutic HBV vaccines have not been evaluated for their efficacy in humans, and those that were typically showed limited efficacy (90–93). These observations clearly stress the need to assess vaccine efficacy in chronic HBV-infected patients. The HBsAg-based prophylactic vaccine is highly effective in preventing the disease mainly by the induction of neutralizing antibodies, while the vaccine failed to induce HBsAg clearance in chronic HBV-infected patients (94–96). These vaccines could have failed to induce a functional cure because they were not potent enough to induce HBV-specific T cells. Additionally, the limited effect of the HBsAg-based prophylactic vaccine, when used in therapeutic strategies, can—at least in part—be explained by the already high levels of circulating HBsAg in chronic HBV patients (97). The observation that therapeutic vaccination against LCMV is more effective when it is administrated in mice with a low viral antigen load may be in line with this (12). Such studies are rather difficult to perform in humans as current NA treatment regimens do not significantly lower HBsAg serum levels (70). More recent studies have evaluated other and additional HBV antigens, such as the core and polymerase protein, and optimized the mode of antigen administration through recombinant antigen- or DNA vaccination (90–92). To date, the majority of therapeutic vaccines have had limited success in clinical trials and induced only a partial restoration of T cells at best, without a durable effect on HBsAg and HBV DNA. A list of previous and therapeutic vaccines under development is provided elsewhere (98–100).

The limited efficacy of current therapeutic vaccines raises the question whether they fail to rejuvenate pre-existing HBV-specific T cells because of ongoing exposure to viral antigens. Alternatively, the vaccine antigens may not sufficiently match the patient's HBV genotype. Given the lack of T cell recovery it seems that these vaccines need to be given in combination with other therapies. In order to enhance their efficacy, vaccination is currently being combined with checkpoint blockade, as these inhibitory receptors may limit clonal expansion of T cells. Indeed, a beneficial effect of PD-1 blockade on therapeutic vaccination is observed in the LCMV mouse model and in woodchucks infected with HBV (101, 102). Moreover, HBV-specific T cells that were induced by dendritic cells are more responsive to PD-1 blockade when compared to those T cells primed by hepatocytes in HBV replication-competent transgenic mice (103). These synergistic effects did, however, not results into a clinical benefit in a small pilot study that administered nivolumab and a therapeutic vaccine to ten virally suppressed chronic HBV patients (83).

Ultimately, these vaccines need to sufficiently reinvigorate antiviral immunity so that hepatocytes infected with HBV can be cleared. This will require a sufficiently broad T cell repertoire covering conserved regions of the virus, so that it can prevent viral escape. One difficulty for the design of epitope-based vaccines is the limited number of HBV class I and II epitopes that have been identified. This is especially relevant as there is a distinct global distribution of HLA alleles and HBV genotypes. Yet the majority of class I epitopes available for human studies are still HLA-A^*^02 restricted and our understanding of genotypic variation of HBV-specific T cell epitopes remains limited (104). The inclusion of degenerate T cell epitopes, i.e., those that can be presented on multiple HLA alleles, could be used to broaden the vaccine efficacy for differing HLA populations. (105, 106).

## Immunotherapy in Different Phases of Chronic Infection

Current guidelines recommend NA treatment for chronic patients with active hepatitis and moderate to severe fibrosis. The inflammatory events and impaired liver architecture of these patients could however be less suited for immunotherapeutic intervention, since they can directly hinder the function of HBV-specific T cells and accessibility to infected hepatocytes (107, 108). In theory, immune tolerant patients could more likely respond to immunotherapy as they have a more preserved liver anatomy and HBV-specific T cells (109). In line with this notion is a recent study that demonstrated that HBV-specific T cells from 13 immune tolerant patients had a significant increase in IFN-γ production in response to overlapping HBV-peptides after the addition IL-2, while this increase could not be observed for 16 immune active patients. (103). Similarly, the fate of T cell exhaustion may be more flexible if the exposure to HBV antigens and other immune impairment mechanisms remains limited. It must be noted that the treatment of immune tolerant patients is still controversial and more detailed studies are needed to further substantiate this hypothesis.

## Future Perspectives

There is extensive evidence indicating that T cells are required for HBV control, but these responses become defective in chronic patients. Immunotherapy aimed at reinvigorating dysfunctional T cells represents a logical approach to induce a functional cure of a chronic infection. Experimental studies can provide a proof of concept, but their efficacy does not always translate into chronic patients. For one, because in chronic patients multiple T cell impairment mechanisms are operative over a period of decades and some T cell defects may not be fully reversible. Recent efforts have begun to better define HBV-specific T cell phenotype and function in relation to their epitope-specificity. Checkpoint inhibitors and therapeutic vaccines have thus far had limited success in a small number of clinical trials, with most studies reporting only a partial recovery of T cells. It must be noted that the optimal drug dosages and the appropriate timing of these treatments are yet to be determined. Additionally, the lack of efficacy has been attributed to the high antigenic burden, in particular of HBsAg, which cannot be overcome by current standard of care HBV therapies. Combining immunomodulation with novel direct-acting antivirals, that can inhibit both viral replication and antigen load may be required to achieve a functional cure. Supported by recent methodological advances of platforms, such as multi-parameter flow cytometry and RNA-sequencing of HBV-specific T cells from both blood and liver it is expected to accelerate progress in the field at an unprecedented level. It is with this increased understanding that we will be able to develop safe and effective immunotherapies for HBV.

## Author Contributions

RH wrote the paper. AB wrote and edited the paper.

### Conflict of Interest

The authors declare that the research was conducted in the absence of any commercial or financial relationships that could be construed as a potential conflict of interest.
